# Inhibition of mycobacteria proliferation in macrophages by low cisplatin concentration through phosphorylated p53-related apoptosis pathway

**DOI:** 10.1371/journal.pone.0281170

**Published:** 2023-01-31

**Authors:** Jiajia Bao, Yonglin He, Chun Yang, Nan Lu, Anlong Li, Sijia Gao, Felycia Fernanda Hosyanto, Jialing Tang, Junzhuo Si, Xia Tang, Huichao Fu, Lei Xu

**Affiliations:** 1 Department of Pathogenic Biology, College of Basic Medicine, Chongqing Medical University, Chongqing, China; 2 Hospital-Acquired Infection Control Department, First People’s Hospital of Jintang County, Chengdu, China; 3 Department of Clinical Medicine, Chongqing Medical University, Chongqing, China; 4 Clinical laboratory, People’s Hospital of Rongchang District, Chongqing, China; Babasaheb Bhimrao Ambedkar University, INDIA

## Abstract

**Background:**

Drug resistance is a prominent problem in the treatment of tuberculosis, so it is urgent to develop new anti- tuberculosis drugs. Here, we investigated the effects and mechanisms of cisplatin (DDP) on intracellular *Mycobacterium smegmatis* to tap the therapeutic potential of DDP in mycobacterial infection.

**Results:**

Macrophages infected with *Mycobacterium smegmatis* were treated with DDP alone or combined with isoniazid or rifampicin. The results showed that the bacterial count in macrophages decreased significantly after DDP (≤ 6 μg/mL) treatment. When isoniazid or rifampicin was combined with DDP, the number of intracellular mycobacteria was also significantly lower than that of isoniazid or rifampicin alone. Apoptosis of infected cells increased after 24 h of DDP treatment, as shown by flow cytometry and transmission electron microscopy detection. Transcriptome sequencing showed that there were 1161 upregulated and 645 downregulated differentially expressed genes (DEGs) between the control group and DDP treatment group. A Trp53-centered protein interaction network was found based on the top 100 significant DEGs through STRING and Cytoscape software. The expression of phosphorylated p53, Bax, JAK, p38 MAPK and PI3K increased after DDP treatment, as shown by Western blot analysis. Inhibitors of JAK, PI3K or p38 MAPK inhibited the increase in cell apoptosis and the reduction in the intracellular bacterial count induced by DDP. The p53 promoter Kevetrin hydrochloride scavenges intracellular mycobacteria. If combined with DDP, Kevetrin hydrochloride could increase the effect of DDP on the elimination of intracellular mycobacteria. In conclusion, DDP at low concentrations could activate the JAK, p38 MAPK and PI3K pathways in infected macrophages, promote the phosphorylation of p53 protein, and increase the ratio of Bax to Bcl-2, leading to cell apoptosis, thus eliminating intracellular bacteria and reducing the spread of mycobacteria.

**Conclusion:**

DDP may be a new host-directed therapy for tuberculosis treatment, as well as the p53 promoter Kevetrin hydrochloride.

## 1. Background

Tuberculosis (TB) is an infectious disease caused by *Mycobacterium tuberculosis* (*M*. *tuberculosis*). In accordance with the estimates in the Global TB Report 2021 published by the WHO in October 2021, there are currently close to 2 billion people with latent TB infection worldwide [1], and 9.87 million new cases were diagnosed in 2020.

Even though the global infection rates have declined slightly, the threat to public health from TB has worsened with the emergence of drug-resistant tuberculosis, particularly multidrug-resistant TB (MDR-TB) and extensively drug-resistant TB (XDR-TB). At present, there are more new cases of MDR-TB, XDR-TB, and even total drug-resistant TB (TDR-TB) [2]. In 2019, there were approximately 500,000 new rifampicin-resistant TB (RR-TB) cases, 78% of which were MDR-TB [3]. The most recent treatment outcome data showed that the success rate for susceptible TB was at least 85%, while the cure rates for MDR-TB and XDR-TB were 50% and 30%, respectively [4]. With the global burden of MDR-TB increasing by more than 20% per year over the past few years, it is estimated that drug-resistant TB will cause more than 70 million deaths and cost the global economy more than US$10 trillion over the next 35 years [5]. It is obviously insufficient to control TB infection and treat all TB patients with traditional anti-TB medications. The development of new TB diagnostics, regimens, and vaccines is imminent. As of September 2021, 25 types of new medications in phase I, II, and III clinical trials were used for TB, comprising 6 kinds of repurposed drugs [1]. Drug repurposing has become a new strategy for drug development as it can reduce drug development costs, reduce regulatory barriers, and improve treatment success rates.

Since their discovery, metal compounds have played a unique role in medicine and diagnosis in addition to their wide use in industry [6–11]. With the increasing number of studies regarding metal compounds in treating infectious diseases, the antibacterial mechanisms include destruction of bacterial cell walls, production of reactive oxygen species (ROS), and disruption of DNA structure [12,13]. Unlike the risk that classical antibiotics are susceptible to developing resistance, metal drugs produce less resistance, which may be related to the fact that metals as antimicrobial agents have a different mechanism of action than conventional antibiotics [14]. Platinum-based drugs were developed in the 1960s, and platinum-based drugs have been in clinical use since 1971; more than 40 years later, platinum drugs remain one of the most widely used anticancer drugs even in the era of precision medicine and immunotherapy. Cisplatin was the first platinum-containing drug to enter clinical trials and was approved for use by the United States Food and Drug Administration in 1978. It is widely used in the therapy of numerous malignant tumors (breast, ovary, head and neck, colorectal, urinary bladder, and neuroblastoma) [15]. In addition to oncological applications, platinum is believed to have broad-spectrum antimicrobial effects, encompassing (1) bacteria, such as *Salmonella* [16], *Staphylococcus aureus* [17], and *Helicobacter* [18]; (2) fungi, e.g., *Candida albicans [19]*; and (3) viruses, such as hepatitis B virus (HBV) [20], hepatitis C virus (HCV) [21]^,^ human immunodeficiency virus (HIV) [22], human cytomegalovirus (HCMV) [23], and herpes simplex virus (HSV) [24]; (4) parasites, for example, malaria [25] and Leishmania [26].

*M*. *tuberculosis* is a highly successful intracellular pathogenic microorganism, and macrophages are its main parasitic site. Consequently, it is necessary to remove intracellular mycobacteria to completely cure TB. Although studies have predicted that platinum compounds may have a site of action against *M*. *tuberculosis* in vitro [27], it is still unclear whether platinum compounds can affect mycobacteria inside the cells. This study aims to investigate the effect of cisplatin on the proliferation of mycobacteria within macrophages and to explore its possible molecular mechanism. According to the new antimycobacterial function of cisplatin, it may be a new way to treat latent infection of *M*. *tuberculosis* and improve the treatment effect of MDR-TB by using it alone or in combination with other antituberculosis medications.

## 2. Materials and methods

### 2.1. Cell culture

The murine macrophage cell line RAW264.7 and the human acute monocytic leukemia cell line THP-1 used in this study were preserved in our laboratory. Murine macrophage J774A.1 cells were purchased from American Type Culture Collection (ATCC, USA). RAW264.7 and J774A.1 cells were cultured in DMEM (HyClone, SH30022.01), which is a high glucose medium containing L-glutamine and no sodium pyruvate. THP-1 cells were cultured in RPMI 1640 medium (HyClone, SH30809.01B),which is a cell medium containing L-glutamine and no calcium nitrate. All media were supplemented with 10% fetal bovine serum (Gibco, 10099–141). All the cells were incubated at 37°C with 5% CO_2_.

### 2.2. *Mycobacterium* culture and infection

The *M*. *smegmatis* (Ms, mc^2^155) used in this study was preserved in our lab. *M*. *smegmatis* strains were revived and cultured in 7H9 medium (supplemented with 0.2% glycerol and 0.05% Tween 80). THP-1, RAW 264.7 and J774A.1 cells were infected with *M*. *smegmatis* at a multiplicity of infection (MOI) of 0.1 at 37°C for 1 h. The infected cells were washed 3 times with PBS to remove the extracellular bacteria and then incubated in fresh medium until the time required for the experiment.

### 2.3. Colony forming unit (CFU) assay

To determine the intracellular bacterial load of macrophages, the infected cells were lysed with 0.5% Triton X-100 at 37°C for 30 seconds. The cell lysate was spread on Middlebrook 7H9 agar plates. The numbers of colonies on the plates were counted after culturing at 37°C for 3 days.

### 2.4. Cell viability and proliferation assays

For cell viability analysis, macrophage cells were seeded into 96-well plates at 5×10^3^ cells per well. Twenty-four hours later, the medium was replaced with fresh medium, and DDP was added to the medium. The doses of DDP were 0, 0.25, 0.5, 1, 2, 4, 8 and 10 μg/mL. The number of viable cells was determined by using a 10 μL cell counting kit-8 (Elabscience Biotechnology, Wuhan, China) assay in an incubator at 37°C with 5% CO_2_ for 1 h. Finally, the absorbance at 450 nm was detected to examine cell viability. The experiment at each concentration was carried out in triplicate. The effect of DDP on cell proliferation was analysed by the trypan blue exclusion assay. Macrophages were seeded at a density of 5 ×10^3^ per well and cultured overnight. After replacing fresh medium, cells were cultured in various concentrations of DDP (0, 0.25, 0.5, 1, 2, 4, 8 and 10 μg/ml). Seventy-two hours after treatment, the attached and floating cells were collected, mixed with an equal volume of 0.4% trypan blue solution (Beyotime, China) for 1 min and counted by a hemocytometer.

### 2.5. RNA extraction

RAW264.7 cells were seeded on 6-well plates (1 × 10^6^ cells/well), incubated overnight in a cell culture incubator at 37°C, and then infected with *M*. *smegmatis* (MOI = 0.1) for 1 h. Then, the cells were washed three times with cell culture medium to remove extracellular mycobacteria, the cells were incubated with medium containing 1 μg/ml DDP for 24 h as the experimental group, and the cells were incubated with cell culture medium supplemented with an equal volume of PBS for 24 h as the control group. For each group, three independent experiments were performed. Total RNA was extracted using TRIzol reagent (Life Technologies, Carlsbad, CA) following the manufacturer’s instructions. The concentration and purity of the extracted RNA were checked by a Nanodrop 2000. The integrity of the RNA was checked by agarose gel electrophoresis, and the Rin values were determined by an Agilent 2100.

### 2.6. mRNA sequencing and bioinformatic analysis

RNA SEQ was performed by Shanghai Meiji Biological Medicine Technology Co., Ltd. Library construction was performed by using the Illumina TruSeq™ RNA Sample Prep Kit. Read counts of each sample transcript were obtained through RSEM, and RPKM conversion was used to eliminate the effect of gene length on calculating gene expression. According to the experimental design, we used the software Cufinks to screen the differentially expressed genes (DEGs) between the DDP-treated group and the control group. We set |Log2(Fold change) |≥ 1 and Q.value<0.05 as the indexes to identify significant differences between the two groups. To further understand the relationship between the DEGs and DDP, we conducted GO analysis and KEGG signaling pathway analysis to predict the potential function of DEGs. GOAtools is a python package that process the obo-formatted file from GO website based on Fisher’s exact test. it maps genes to GO terms efficiently.The R script was used to perform KEGG pathway enrichment analysis of the transcripts in the gene set, and the calculation principle was the same as that of GO functional enrichment analysis. Using *p* value < 0.05 as the standard for functional enrichment analysis.

String database is a database for searching known and predicted protein-protein interactions. Protein-protein interaction network is constructed by integrating previous experimental data, article results from comprehensive databases such as Pubmed and bioinformatics prediction. In our rearch,PPI analysis of the top 100 DEGs was performed by the STRING database, and the results were exported in TSV format and visualized through Cytoscape software.

### 2.7. Flow cytometry

The macrophages were collected and fixed in cold 70% ethanol and stored at 4 °C for 30 min. Ethanol was then removed, and then the cells were washed with PBS twice and digested into single cells with 0.25% trypsin (without EDTA). After washing with PBS again, the cells were stained with DNA staining solution for cell cycle detection or stained with annexin V-FITC/PI double staining for the apoptosis assay. The flow cytometry data were analysed using FlowJo Software.

### 2.8. Western blotting

Cellular proteins were extracted using RIPA reagent (Solarbio, R0010), which was mixed with the phosphatase inhibitor Halt (Solarbio, P1260) and protease inhibitor PMSF (Solarbio, P0100) at a ratio of 100:1:1. Protein concentrations were determined using a BCA protein concentration determination kit (Beyotime Institute of Biotechnology, Shanghai, China). The extracted proteins were electrophoretically separated by SDS–PAGE at 110 V and then transferred onto polyvinylidene fluoride (PVDF) membranes. After blocking with 5% nonfat milk for 2 h at room temperature, the membranes were incubated with primary antibodies overnight at 4°C. The primary antibodies used in the study included rabbit anti-mouse p-p53 (1:500, Huaan Biological, China), rabbit anti-mouse p38 MAPK protein (1:1000, Zhengneng Biological, China), rabbit anti-mouse JAK protein (1:1000, Zhengneng Biological, China), rabbit anti-mouse PI3K protein (1:1000, Zhengneng Biological, China), rabbit anti-mouse Bax protein (1:500, Huaan Biological, China), rabbit anti-mouse Bcl-2 protein (1:500, Huaan Biological, China) and rabbit anti-mouse GAPDH protein (1:2000, Sanying Biological, China). The membranes were washed four times for 10 min with TBST and incubated with the appropriate HRP-conjugated Alpaca anti-rabbit IgG antibody (1:5000, Huaan Biological, China) for 1 h at 25°C. The washing step was repeated, and the target proteins were visualized and quantified with a Biohypersensitive Rad ECL chemiluminescence kit (NCM Biotech, P2300).

### 2.9. Transmission electron microscopy (TEM)

RAW264.7 cells were collected, and a compact sample mass was formed at the bottom of the tube after centrifugation at 1200 rpm for 5 min. Then, the supernatant was discarded, and 2.5% glutaraldehyde fixing solution was gently added along the tube wall. The specimen was embedded in pure epoxy resin after gradient acetone dehydration. The embedded material was cut into 70 nm ultrathin sections and then dyed with lead citrate dye containing uranium acetate (3%) for photography under a transmission microscope.

### 2.10. Statistical analysis

All statistical analyses were performed by using GraphPad Prism Software Version 8.0 (Grapad Prism Software, La Jolla, CA). All data in the study are presented as the mean ± standard deviation from at least three independent experiments. Statistical comparisons were made by unpaired Student’s t test (for two group comparisons) or one-way analysis of variance (for multiple group comparisons). *p* < 0.05 was considered significantly different.

## 3. Results

### 3.1. Removal of *M*. *smegmatis* from macrophages by low concentrations of DDP alone or combined with first-line anti-tuberculosis drug*s*

**Fig 1:**
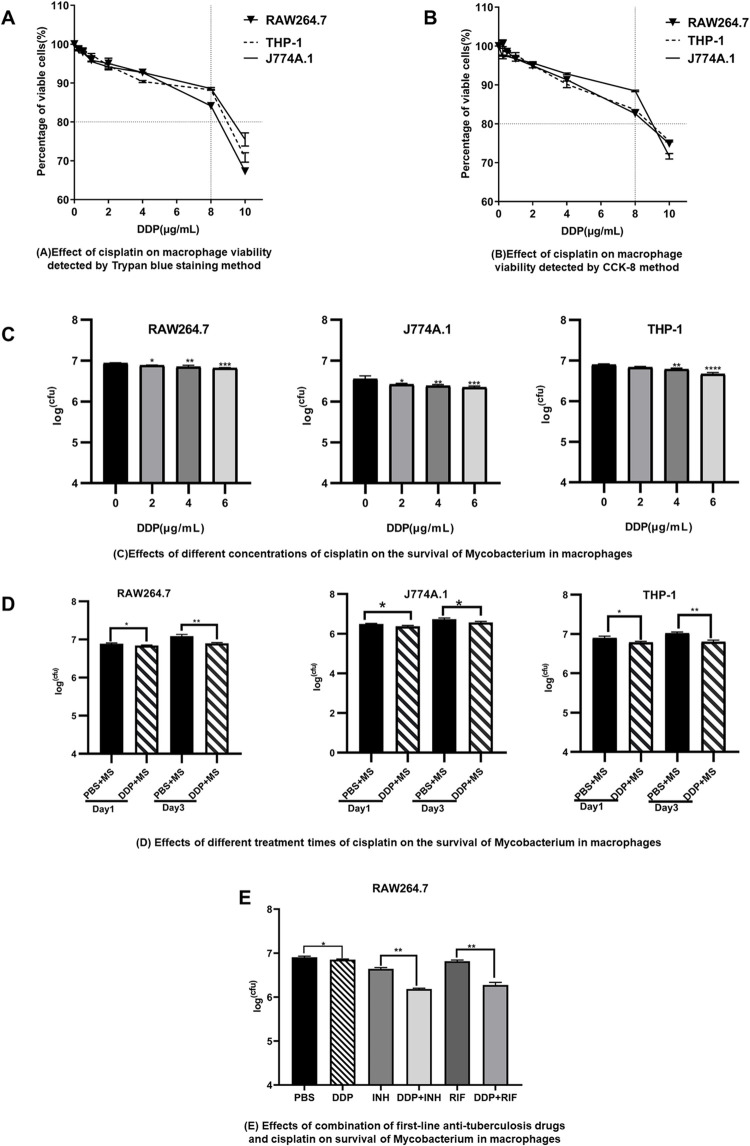
Elimination of *M*. *smegmatis* from macrophages by low concentrations of DDP alone or combined with first-line anti-tuberculosis drugs. (A) The effect of DDP on cell proliferation by the trypan blue exclusion assay. The doses of DDP were 0, 0.25, 0.5, 1, 2, 4, 8 and 10 μg/mL. (B) The effect of DDP on cell viability by CCK-8 (cell counting kit-8) detection. (C) CFU of *intracellular M*. *smegmatis* after treatment with different concentrations of DDP. Macrophage cell lines J774A.1, RAW264.7, and THP-1 were infected with *M*. *smegmatis* at an MOI of 0.1 for 1 hour, and then the cells were treated with DDP for 24 hours. The doses of DDP were 0, 2, 4, and 6 μg/mL. (D) CFU of *intracellular M*. *smegmatis* after DDP treatment for different times. Macrophages were infected with *M*. *smegmatis* at an MOI of 0.1 for 1 hour, and then cells were treated with 1 μg/mL DDP or PBS for 1 day and 3 days, respectively. (E) CFU of *intracellular M*. *smegmatis* after DDP treatment alone or combined with isoniazid (INH) or rifampicin (RIF). RAW264.7 cells were infected with *M*. *smegmatis* at an MOI of 0.1 for 1 hour and then treated with DDP or INH, RIF, DDP combined with INH, or DDP combined with RIF for 24 hours. The concentrations of DDP, INH and RIF were 1μg/mL, 5 μM and 10 μM, respectively. The experiment for each group was carried out in triplicate. * *p* < 0.05, **p < 0.01, ***p < 0.001, ****p < 0.0001. MS: *M*. *smegmatis*; DDP: Cisplatin.

The trypan blue exclusion assay (Fig 1A) and CCK8 assay (Fig 1B) showed that when the concentration of DDP was lower than 6 μg/mL, the cell viability of the three kinds of macrophages (J774A.1, RAW264.7 and THP-1) was greater than 85%. This indicated that DDP at a concentration of no more than 6 μg/mL had little toxicity to macrophages and was considered a low concentration of DDP. Therefore, we chose a low concentration of DDP for the experiment. Compared with the PBS group, the number of *M*. *smegmatis* in macrophages of the DDP group decreased significantly, and the degree of reduction was dose-dependent with DDP (Fig 1C). Treatment with 1 μg /mL DDP for 1 day inhibited the survival of mycobacteria in the cells, and this effect was further increased on the third day of DDP treatment (Fig 1D). These results showed that the clearance of Mycobacterium in macrophages by DDP was also time dependent. When INH or RIF was combined with DDP, the bacterial count in macrophages was decreased compared with INH or RIF alone, and the difference was statistically significant (Fig 1E).

### 3.2. Low concentrations of DDP eliminate *M*. *smegmatis* in macrophages by promoting apoptosis

**Fig 2:**
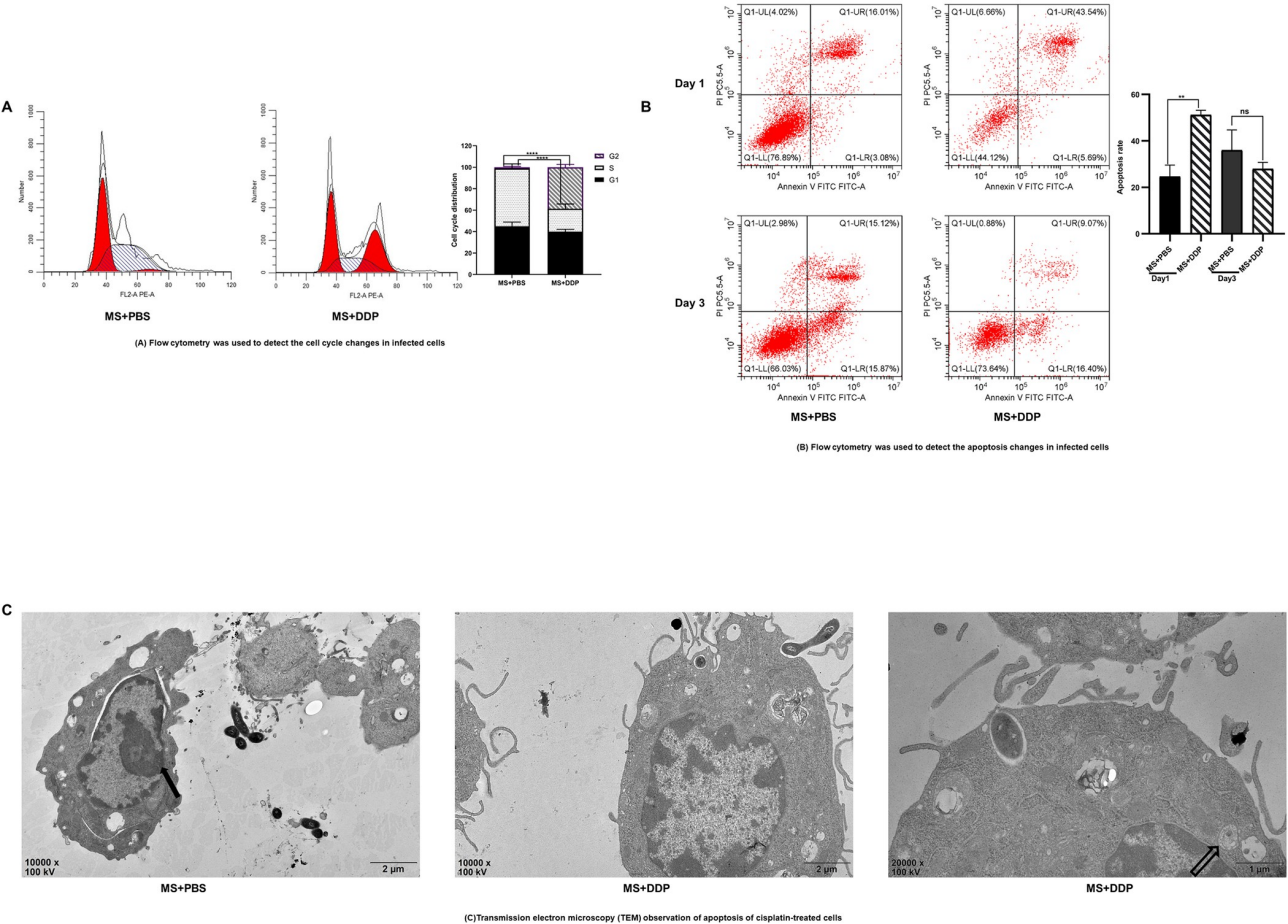
Low concentrations of DDP eliminate intracellular *M*. *smegmatis* by promoting apoptosis. (A) Cell cycle arrest in the G2 phase after DDP treatment by flow cytometry analysis. RAW264.7 cells were infected with *M*. *smegmatis* at an MOI of 0.1 for 1 hour and then treated with 1 μg/mL DDP or PBS for 24 h. (B) Cell apoptosis was detected by flow cytometry after DDP treatment. RAW264.7 cells infected with *M*. *smegmatis* were treated with 1 μg/mL DDP or PBS for 1 day and 3 days, respectively. (C) Cell apoptosis was detected through TEM observation. RAW264.7 cells infected with *M*. *smegmatis* were treated with 1 μg/mL DDP or PBS for 24 h. The filled arrow indicates the intact nuclear membrane, and the white arrow indicates the apoptotic bodies. * *p* < 0.05, **p < 0.01. MS: *M*. *smegmatis*; DDP: Cisplatin.

RAW264.7 cells were infected with *M*. *smegmatis* mc^2^155 at an MOI of 0.1 for an hour and then treated with DDP (1 μg /mL) or PBS for another 24 h. Flow cytometry analysis revealed that DDP caused cell cycle arrest in the G2 phase (Fig 2A), and the apoptosis rate of macrophages was significantly increased in the DDP group compared with the PBS group (p<0.01) (Fig 2B). After 72 h of treatment, the cell apoptosis rate was 27. 94 ± 2.79 in the DDP-treated group and 35. 98 ± 8.74 in the control group, without a significant difference (Fig 2B).

Macrophages infected with *M*. *smegmatis* had a certain degree of apoptosis based on TEM observation. Under a single random field, the number of apoptotic cells in the DDP treatment group was greater than that in the control group. At a magnification of 10000x, although the cell membrane of the PBS group did not form a regular circle, the nuclear membrane remained intact. However, the apoptotic phenomenon was more obvious in the DDP-treated group, with obvious apoptotic bodies surrounded by double-layer membranes. The cell volume decreased, the cytoplasmic density increased, the mitochondrial matrix was compact, nuclear division was obvious, and the lobulated nuclei were irregular (Fig 2C).

### 3.3. Results of transcriptome sequencing and bioinformatics analysis

**Fig 3:**
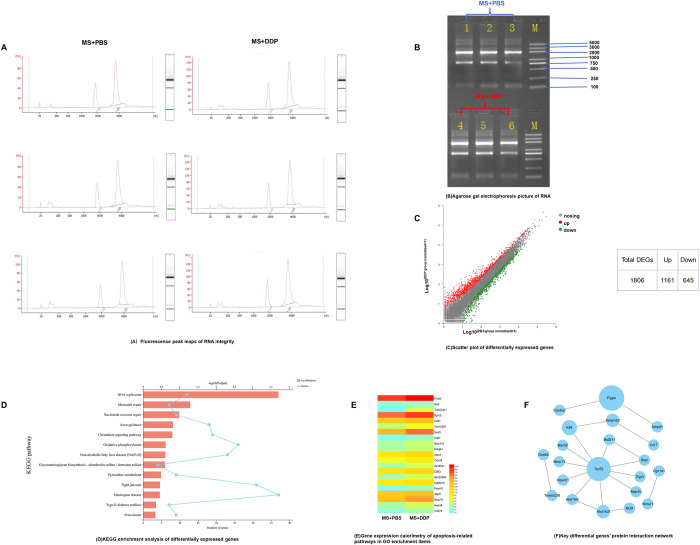
Results of transcriptome sequencing and bioinformatics analysis. RAW264.7 cells were infected with *M*. *smegmatis* at an MOI of 0.1 for 1 hour and then treated with 1 μg/mL DDP or PBS for 48 h. (A) Detection of sample RNA integrity by agarose gel electrophoresis. (B) A scatter plot of DEGs, including 1161 upregulated DEGs and 645 downregulated DEGs. (C) Pathway enrichment of DEGs by KEGG. (D) The heatmap of the expression of DEGs with apoptosis-related pathways by Goatools. (E) The protein interaction network of the top 100 significant DEGs through STRING and Cytoscape software.

To further understand the mechanism of DDP on infected macrophages, 1 μg/mL DDP or PBS was used to treat *M*. *smegmatis-*infected macrophages for 48 h, and then the total RNA was subsequently extracted from the samples. The RNA integrity number (RIN, the normal range is 1–10, and the higher the value is, the better the RNA integrity) of each sample detected by agarose gel electrophoresis was 10 (Fig 3A), Agarose gel electrophoresis picture of RNA is shown in Fig 3B. Transcriptome sequencing of 6 samples (including 3 DDP-treated groups and 3 PBS-treated groups) was completed, and 43.87 GB of clean data was obtained. The clean data of each sample were more than 6.8 GB, and the percentage of the Q30 base was more than 96.05% (Table 1). In total, 1161 upregulated DEGs and 645 downregulated DEGs were obtained, and a scatter plot was drawn (Fig 3C). DEGs were mainly clustered in DNA replication, damage repair, nuclear mismatch repair, cell cycle regulation, oxidative stress and other pathways by KEGG analysis (Fig 3D). A heatmap (Fig 3E) was drawn based on the gene expression data of apoptosis-related pathways in GO enrichment entries, and Bad and Trp53cor1 ranked top in the heatmap. A Trp53 (Trp53 is considered p53 in the mouse genome)-centered protein interaction network was found based on the top 100 significant DEGs through STRING and Cytoscape software (Fig 3F).

**Table 1:** Sequencing data statistics.

Sample number	Clean reads	Error rate(%)	Q20(%)	Q30(%)
**C1**	53971518	0.0231	98.79	96.05
**C2**	47787808	0.0229	98.87	96.29
**C3**	55252244	0.023	98.83	96.14
**D1**	46416326	0.0231	98.81	96.13
**D2**	48033538	0.023	98.83	96.25
**D3**	49263548	0.0231	98.81	96.16

C: Control group; D: DDP group.

### 3.4. Low concentrations of DDP promote p53 phosphorylation to eliminate *M*. *smegmatis* in macrophages

**Fig 4:**
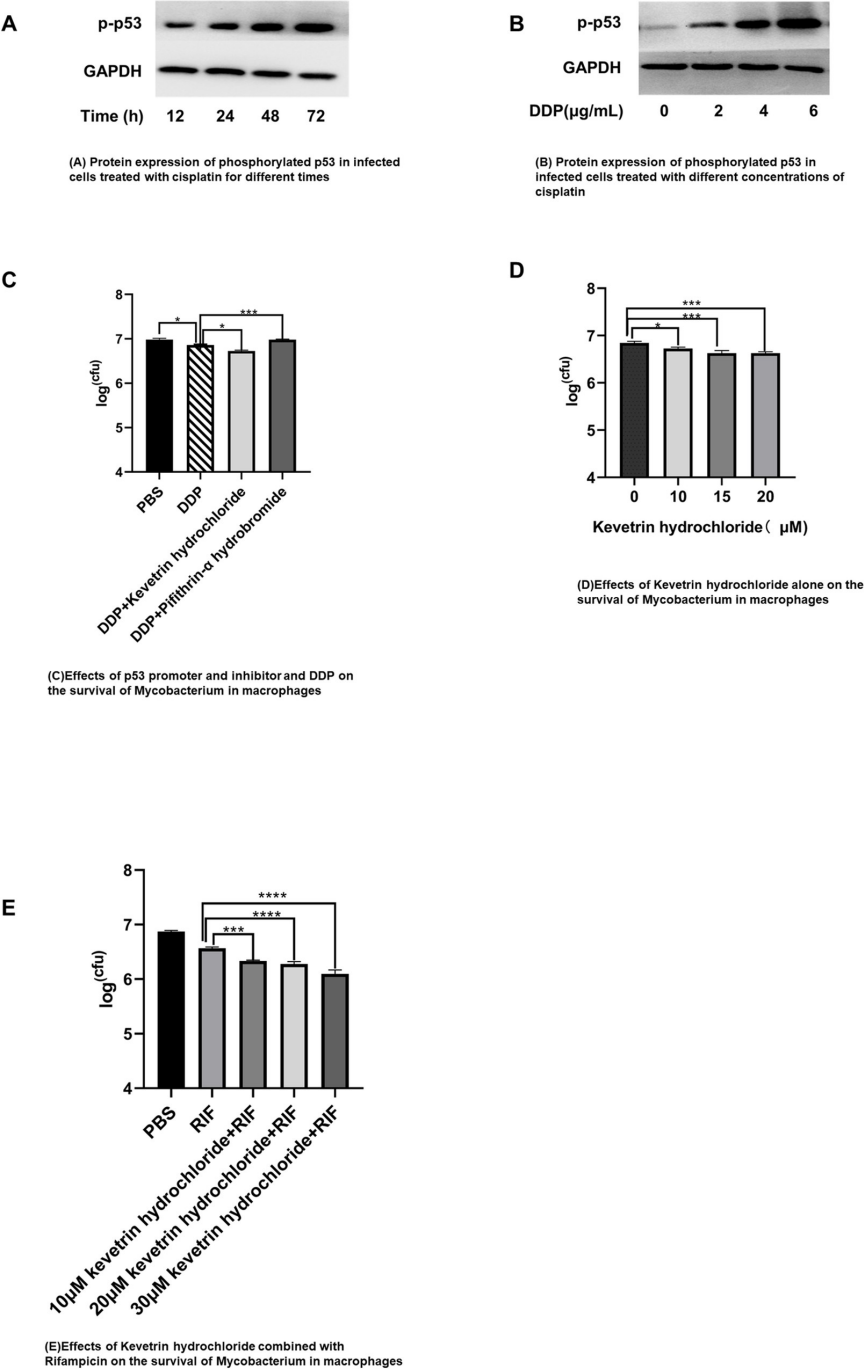
Low concentration of DDP promotes p53 phosphorylation to eliminate *M*. *smegmatis* in macrophages. (A) Western-blot detection the expression of phosphorylated p53 after 1 μg/mL DDP treatment for different time. (B) Western-blot detection phosphorylated p53 expression 48 h after DDP treatment at different concentration. (C) Bacteria count in macrophages after the treatment of DDP combined with p53 promoter or inhibitor. Raw264.7 cells infected with *M*.*smegmatis* were treated with 1 μg/mL DDP, or 1 μg/mL DDP combined with 10 μM p53 promoter (Kevetrin hydrochloride), or 1 μg/mL DDP combined with 10 μM p53 inhibitor (Pifithrin -α hydrobromide) for 24 h. (D) CFU in macrophages after Kevetrin hydrochloride treatment. Raw264.7 cells infected with *M*.*smegmatis* were treated with Kevetrin hydrochloride at the concentration of 10 μM, 15 μM, 20 μM for 24 h respectively. (E) CFU in macrophages after Kevetrin hydrochloride treatment alone or combined with RIF. **p < 0.01, ***p < 0.001, ****p < 0.0001. MS:*M*.*smegmatis*; DDP:Cisplatin.

The results of Western blot detection showed that the expression of phosphorylated p53 protein increased after DDP treatment, and this effect was positively correlated with the concentration and the time of DDP treatment (Fig 4A and 4B). The CFU by spread plate method showed in DDP combined with p53 promoter (Kevetrin hydrochloride) group decreased significately compared with DDP group (*p*<0.0001), while the number of colonies in DDP combined with p53 inhibitor (Pifithrin -α hydrobromide) group increased greatly compared with DDP group (*p*<0.0001) (Fig 4C). Moreover, the bacterial number in cells could also be significantly reduced by using Kevetrin hydrochloride alone, and this effect was positively correlated with the concentration of the p53 promoter (Fig 4D). Compared with RIF, RIF combined with Kevetrin hydrochloride had a stronger effect on the elimination of intracellular mycobacteria (Fig 4E).

### 3.5. Low concentrations of DDP eliminate intracellular *Mycobacteria* by activating phosphorylated p53 through the JAK, PI3K and p38 MAPK pathways

**Fig 5:**
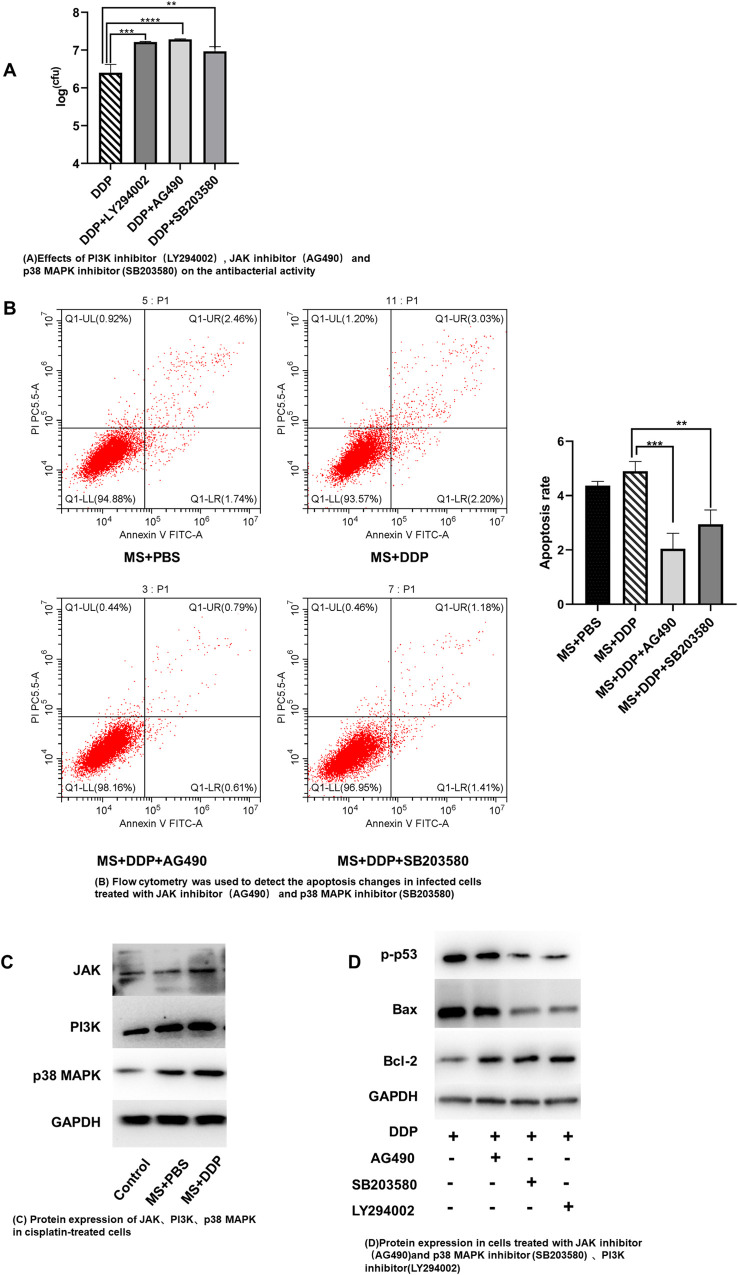
Inhibition of mycobacteria proliferation in macrophages by low cisplatin concentration through phosphorylated p53-related apoptosis pathway. (A) Bacteria count in macrophages after DDP treatment alone or combined with 10 μM JAK inhibitor (AG490) or 50 μM PI3K inhibitor (LY294002) or 5 μM p38 MAPK inhibitor (SB203580) for 12 h. (B) The cell apoptosis after DDP treatment alone or combined with JAK inhibitor or PI3K inhibitor or p38 MAPK inhibitor for 12 h through the Flow cytometry analysis. (C) The expression of JAK, p38 MAPK and PI3K increased after DDP treatment for 12 h by Western-blot analysis. (D) The expression of phosphorylated p53, Bax and Bcl-2 in macrophages after the treatment of DDP combined with 10 μM JAK inhibitor (AG490) or 50 μM PI3K inhibitor (LY294002) or 5 μM p38 MAPK inhibitor (SB203580) respectively through Western-blot. **p < 0.01, ***p < 0.001, ****p < 0.0001. MS:*M*.*smegmatis*; DDP:Cisplatin.

Compared with the DDP-treated group, the groups treated with DDP combined with JAK inhibitor (AG490), PI3K inhibitor (LY294002) or p38 MAPK inhibitor (SB203580) showed significantly increased bacterial counts in macrophages (Fig 5A). The addition of AG490, LY294002 or SB203580 reduced the apoptosis rate induced by DDP, as shown by flow cytometry analysis (Fig 5B). The expression of JAK, p38 MAPK and PI3K increased after DDP treatment, as shown by Western blot analysis (Fig 5C). Compared with DDP treatment, the expression of phosphorylated p53, Bax decreased and Bcl-2 increased in the DDP combined with AG490, SB203580 or LY294002 groups, respectively, according to Western blotting. Compared to the DDP group, the ratio of Bax/Bcl-2 reflecting the level of apoptosis decreased in the DDP combined with AG490, SB203580 or LY294002 groups (Fig 5D).

## 4. Discussion

*M*. *tuberculosis* has coevolved with its hosts for hundreds or thousands of years and can survive within phagocytes for a long time. Therefore, cellular immunity plays an extremely remarkable role in the pathogenesis and prognosis once *M*. *tuberculosis* invades the human body. As a part of cellular immunity, macrophages are the first-line defense against *M*. *tuberculosis* [28] and play a central role in the pathogenesis of this bacteria [29] in addition to the parasitic sites of *M*. *tuberculosis* within the body. Macrophages eliminate *M*. *tuberculosis* through multiple mechanisms [30], including 1) the production of oxygen and nitrogen to promote phagosome acidification and phagosome-lysosomal membrane fusion and the production of granzymes, granulysin, and perforin to kill *M*. *tuberculosis*; 2) the secretion of inflammatory cytokines, including IFN-γ, IL-12, IL-1β and macrophage inflammatory protein-1α (MIP-1α/CCL3) to resist Mtb infection; 3) the cessation of microorganism dissemination by apoptosis; 4) the stimulation of T-lymphocyte responses by antigen presentation promotes elimination; 5) after activation, autophagosome production by macrophages will transport *M*. *tuberculosis* to lysosomes for degradation and enhance destruction.

In most cases, however, intracellular *M*. *tuberculosis* is not completely eliminated and replicates in macrophages since *M*. *tuberculosis* utilizes a number of ingenious strategies to evade the host immune response [6]. This leads to the retention or proliferation of *M*. *tuberculosis* and even granuloma formation. The pathophysiology of tuberculosis highly depends on the ability of mycobacteria to disrupt the innate immune response of macrophages [31]. The complex interaction between *M*. *tuberculosis* and host macrophages is also involved in the development of resistance to *M*. *tuberculosis* and the persistence of bacterial infection, while manipulating the macrophage response to *M*. *tuberculosis* has been suggested as a promising new pathway for the treatment and vaccine development of TB [31,32].

Apoptosis is also known as programmed cell death. Numerous studies have shown that apoptosis promotes the elimination of intracellular pathogens, including bacteria, fungi, viruses, and parasites [33–43]. During apoptosis, the cytoplasm shrinks, chromatin condenses into a crescent shape, the nucleus is fragmented, and the cell membrane wraps the cell components to form apoptotic bodies, which are then phagocytosed. Thus, the intracellular components will not be released outside the cell. Apoptosis does not lead to host immune cascade amplification; that is, apoptosis does not induce inflammation, nor does it result in the depletion of cytokines that result in cytokine release. Therefore, apoptosis is considered to be a beneficial defense response beneficial to the host. The possible mechanisms by which apoptosis promotes the elimination of intracellular microorganisms [44] include 1) preventing the release of intracellular pathogens, reducing the dissemination of pathogens [45] or even dissemination between different species [46]; 2) absorption of apoptotic bodies, as antigen reservoirs, by phagocytes through receptor-mediated phagocytosis, which are degraded and presented as MHC class II complexes to promote the initiation and response of T-lymphocyte immunity [47]; and 3) apoptosis helps to reduce the viability of pathogens and directly affects the host–pathogen balance for infection control [48].

Our study found that although a high concentration of DDP can kill *M*. *smegmatis* directly, this high concentration can also cause a large amount of cell death; thus, it is not a feasible solution for the treatment of mycobacteria infection. Therefore, we chose low concentrations of DDP (concentrations with no apparent cytotoxicity) for our study. Fortunately, low concentrations of DDP had a significant inhibitory effect on the proliferation of *M*. *smegmatis* in macrophages. The results of flow cytometry, electron microscopy, and apoptotic protein expression suggested that the above phenomenon might be achieved by promoting cell apoptosis. Interestingly, in the early stage (24 hours) of DDP treatment, the number of cells in the drug group decreased due to apoptosis. However, in the middle and late treatment periods (72 hours), the number of cells in the drug group increased significantly compared with that in the control group (nondrug treatment group), and the ratio of apoptosis was significantly lower than that in the control group. The reason might be that the increase in the number of bacteria in the control group led to accelerated cell apoptosis and necrosis. In the drug-treated group, the cells infected with bacteria were already apoptotic, providing space and conditions for noninfected cells to proliferate, and healthy cells proliferated rapidly and completely replaced the infected cells. Presumably, in both uninfected and infected cells, low concentrations of DDP appear to be more prone to induce apoptosis in *M*. *smegmatis*-infected cells, creating a favorable environment for uninfected macrophage proliferation. This is consistent with the experimental results of Srinivasan L et al [44], which further confirms that the use of reasonable pro-apoptotic means may facilitate the complete elimination of *M*. *tuberculosis* after infection and restore health.

The interaction of multiple signal transduction and signaling pathway cascades is involved in apoptosis. It can be divided into the endogenous mitochondrial pathway and exogenous death receptor pathway according to the pathway of occurrence. p53, known as a tumor suppressor protein, controls the cell cycle and cell division during tumor growth by promoting apoptosis and DNA repair [49,50]. Under stress conditions, p53 induces p21 expression and mediates cell cycle arrest [51]. When the damage cannot be repaired, p53 regulates the molecules involved in the apoptosis death receptors (exogenous) and mitochondrial-dependent (endogenous) pathways, mediating apoptosis from both endogenous and exogenous pathways, with both pathways leading to the activation of caspase signaling [52], particularly in the mitochondrial pathway, with p53 interacting with Bcl-2 family proteins, and its downstream proteins Bax and Bid are the most important apoptosis-related genes.

In infectious diseases, p53 plays a prominent role in the elimination of pathogens from the host and the outcome after infection. *Helicobacter pylori* infection reduces the stability of p53 under stress by reducing the expression of the transcription factor USF1, leading to the progression of infection towards gastric cancer [53]. By means of the E6 protein, high-risk human papillomavirus (HPV) induces proteasome-dependent degradation of p53, disrupts DNA repair mechanisms, lessens apoptosis, and causes persistent infection of the virus, which will cause cervical cancer [54]. Inhibition of p53/NF-κB signaling by mycoplasma infection may also lead to persistent infection and cell carcinogenesis [55]. Yann Breton et al. found that knockdown of p53, knockdown of p21, or the use of p53 siRNA and other methods to attenuate the effect of p53 increased the infection rate of human HIV-1 in macrophages [56]. Moreover, liver biopsy samples from patients with chronic hepatitis B (CHB) with high viral loads were found to be significantly different from those with low viral loads, viz. Decreased p53 levels and increased p53 levels with butyrate or inhibition of SIRT-1 can reduce HBV-DNA and hepatitis B surface antigen (HBsAg) [57]. Avian leukosis virus subgroup J (ALV-J) infection can lead to severe immunosuppression, resulting in the occurrence of avian multiple organ tumors. Stimulation of p53 upon ALV-J infection reduces the suppression of innate immune responses in host cells and induces cell cycle-related gene p21 to cause G1 and G2 phase arrest, affecting the expression of apoptosis-related genes Bcl-2 and bak and oncogene c-myc to promote cell apoptosis to inhibit ALV-J replication [58]. Porcine epidemic diarrhea virus (PEDV) can cause porcine epidemic diarrhea with high lethality in piglets. Studies have found that overexpression of p53 or the use of p53-specific activators can lead to the activation of IFN activating elements ISRE, increased release of IFN-stimulated gene (ISG) and IFN-β, significantly reducing viral replication, and knockout or inhibition of P53 will result in increased viral replication [59]. In addition to viruses, p53 restriction of intracellular bacterial [60,61], fungal [62,63], and parasitic [61,64] infections has also been reported.

In a study of mycobacterial infection, p53-deficient macrophages exhibited lower rates of apoptosis and increased intracellular mycobacterial survival. Lim Y et al. found that the p53 promoter Nutlin-3 effectively eliminated the intracellular survival of *M*. *tuberculosis* after infecting macrophages of TB patients and healthy controls with H37Ra for 24 hours [65]. In this study, we found that after DDP treatment, although the expression of p53 in macrophages did not significantly increase, the level of pp53 significantly increased, suggesting that DDP may mediate apoptosis by promoting p53 phosphorylation, thereby inhibiting the proliferation of intracellular *M*. *smegmatis*. In addition, we also found that the proliferation of mycobacteria was obviously inhibited or promoted by adding the promoter or inhibitor of p53 to the cell culture medium. After the addition of the p53 inhibitor, flow cytometry showed that the apoptosis of macrophages was inhibited so that mycobacteria could continue to survive and the number of colonies increased, but vice versa for the promoter. It can be speculated that p53 can effectively inhibit the intracellular survival of mycobacteria in the host, and p53 may become a new target for TB treatment. Meanwhile, low-concentration DDP probably exerts its anti-tuberculosis effect through p53 phosphorylation-mediated apoptosis.

Signal transducer and activator of transcription, STAT, is activated by Janus kinase (JAK) following phosphorylation and transported to the nucleus through the nuclear membrane after dimerization, playing the role of cytokine signal transduction and regulation of specific gene transcription. This JAK/STAT signaling pathway is involved in various physiological activities, such as cell proliferation, differentiation, apoptosis, and immune regulation [66]. The activation of IFN-β-JAK/STAT can stabilize the activities of NF-κb and IRF3, putting the host into an antiviral state and promoting apoptosis [67].

p38MAPK is a MAP kinase that is activated in macrophages during stress. Activated p38MAPK is responsible for protein phosphorylation and the activation of numerous transcription factors, including HSP27, TNF-α, IL-1β, and CHOP. The p38MAPK pathway is involved in the infection and immune regulation of various pathogenic microorganisms, i.e., *Yersinia enterocolitica* [68], *Staphylococcus aureus* [69], *Candida albicans*, and infectious salmon anemia virus (ISAV) [70]. During infection, these microorganisms protect themselves from the immune responses of the host by regulating p38MAPK levels in host cells (including macrophages). The activation of p38MAPK is associated with increased apoptosis levels and increased cytokine release [71].

**Fig 6:**
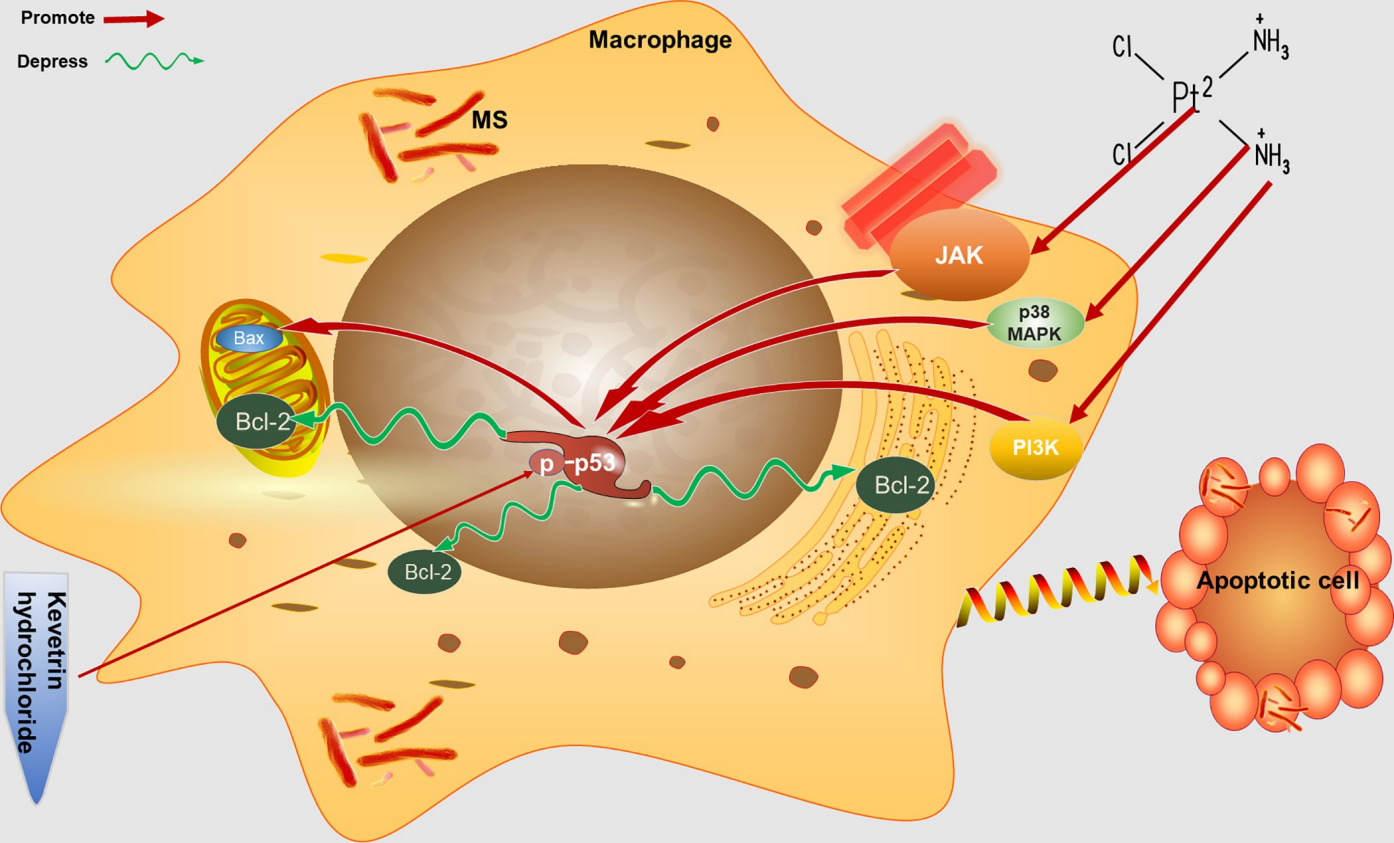
The mechanisms of DDP at low concentration elimination intracellular Mycobacterium. Low concentration of DDP activates JAK, p38 MAPK and PI3K pathways in the infected macrophages, promotes the phosphorylation of p53 protein, and then the expression of apoptosis related protein Bax increased and Bcl-2 decreased, leading to the cell apoptosis, thus eliminating intracellular mycobacteria and reducing the spread of mycobacteria. MS:*M*.*smegmatis*; DDP:Cisplatin.

p38MAPK-mediated phosphorylation of p53 is a critical step for the induction of apoptosis by pathogens such as HIV-1 [72], transmissible gastroenteritis virus (TGEV) [73], and *Clostridium difficile* [74]. Furthermore, it was found that macrophages infected with pathogenic mycobacteria were restricted in the activation of the p38MAPK pathway compared with nonpathogenic mycobacteria [75]. Therefore, the ability to restrict p38MAPK activity may be one of the mechanisms of mycobacterial immune escape. The PI3K pathway plays a role in tumors and infectious diseases by participating in various biological activities, such as cell proliferation, apoptosis, and inflammation. Mutation of PI3K is closely related to uncontrolled apoptosis and tumorigenesis. The upregulation of numerous immune cytokines (granulocyte-macrophage colony-stimulating factor (GM-CSF)) [76], white blood cell agglutinin-1 (Lkn-1) [77,78], and IL-17 [79] that are caused by mycobacterial infection are mediated through PI3K-associated signalling pathways. For active TB patients, the PI3K signaling pathway in immune cells is impaired [80]. Our study found that DDP treatment increased the expression of JAK, p38 MAPK and PI3K proteins, and when the JAK, p38 MAPK or PI3K pathway was inhibited, the effect of DDP could be reversed, resulting in an increase in mycobacteria within macrophages. The possible reason is that inhibiting the above pathway can downregulate the level of phosphorylated p53 protein, reduce Bax/Bcl-2, and reduce apoptosis. The results suggest that DDP is involved in the elimination of intramacrophage mycobacteria through the apoptotic pathway, with p53 and its upstream molecules JAK, p38 MAPK, and PI3K all involved in this process (Fig 6).

In 2020, 71% of TB patients worldwide were resistant to rifampicin, but the development of new antibacterial drugs against *M*. *tuberculosis* has been very slow in the past 10 years. While developing new anti-tuberculosis drugs, reutilization of drugs is an option for controlling drug resistance in TB [81]. The mechanism of action of the currently used first-line anti-tuberculosis drugs is to inhibit the synthesis of mycobacterial DNA and RNA and to hinder the synthesis of mycobacterial acids, fatty acids, and cell walls. However, host-directed therapy (HDT) modulates the biological pathways related to host immunity by promoting macrophage autophagy, apoptosis, the production of ROS, antimicrobial peptides, etc., to change the effect of infected cells on pathogens [82]. Combining HDT with traditional antimicrobial therapy can control the infection faster, shorten treatment time, modulate inflammatory response, and ultimately reduce tissue damage.

Although we initially were concerned that the pro-apoptotic effects of DDP would kill healthy cells, it was later found that low concentrations of DDP (≤ 6 μg/mL) did not significantly damage macrophages without infection with bacteria but had a pro-apoptotic effect on macrophages infected with *M*. *smegmatis*. This may be due to the macrophages infected with bacteria initiating a certain degree of apoptosis, and DDP accelerated or enhanced this process, reducing the cell death caused by mycobacteria proliferation. In our experiment, we also found that when treated with DDP for 72 hours, the number of cells in the drug group increased significantly compared with that in the control group, and the number of intracellular bacteria was significantly reduced. Additionally, DDP exhibited synergistic antimycobacterial effects when used in combination with isoniazid or rifampicin. The p53 promoter Kevetrin also reduced *M*. *smegmatis* in macrophages. These results suggest that DDP and its apoptosis-related signaling molecules may be a new HDT strategy for TB treatment.

## 5. Conclusions

Overall, the main finding of the present work shows that a low concentration of DDP (≤ 6 μg/mL) with little cytotoxicity could eliminate intracellular bacteria and reduce the spread of mycobacteria through cell apoptosis. DDP may activate the JAK, p38 MAPK and PI3K pathways in infected macrophages and then promote the phosphorylation of p53 protein, which could change the expression level of the apoptosis-related proteins Bax and Bcl-2 and lead to cell apoptosis. Therefore, DDP may be a new host-directed therapy for TB treatment, and it also provides ideas for the treatment of other intracellular pathogens.

## Abbreviations

DDP: cisplatin
TB: Tuberculosis;M.tuberculosis:Mycobacterium tuberculosis
MDR-TB: multidrug-resistant TB
XDR-TB: extensively drug-resistant TB
TDR-TB: total drug-resistant TB
RR-TB: rifampicin-resistant TB
ROS: reactive oxygen species
HBV: hepatitis B virus;HCV:hepatitis C virus
HIV: human immunodeficiency virus
HCMV: human cytomegalovirus;HSV:herpes simplex virus;INH:isoniazid
RIF: rifampicin
DEGs: differentially expressed genes
ATCC: American Type Culture Collection
MOI: multiplicity of infection;CFU:Colony forming unit
TEM: Transmission electron microscopy
